# Importance of the postcranial skeleton in eusuchian phylogeny: Reassessing the systematics of allodaposuchid crocodylians

**DOI:** 10.1371/journal.pone.0251900

**Published:** 2021-06-09

**Authors:** Alejandro Blanco

**Affiliations:** 1 Centro de Investigacións Científicas Avanzadas (CICA), Facultade de Ciencias, Universidade da Coruña, A Coruña, Spain; 2 Bayerische Staatssammlung für Paläontologie und Geologie, München, Germany; Chinese Academy of Sciences, CHINA

## Abstract

Our current knowledge on the crocodyliform evolution is strongly biased towards the skull morphology, and the postcranial skeleton is usually neglected in many taxonomic descriptions. However, it is logical to expect that it can contribute with its own phylogenetic signal. In this paper, the changes in the tree topology caused by the addition of the postcranial information are analysed for the family Allodaposuchidae, the most representative eusuchians in the latest Cretaceous of Europe. At present, different phylogenetic hypotheses have been proposed for this group without reaching a consensus. The results of this paper evidence a shift in the phylogenetic position when the postcranium is included in the dataset, pointing to a relevant phylogenetic signal in the postcranial elements. Finally, the phylogenetic relationships of allodaposuchids within Eusuchia are reassessed; and the internal relationships within Allodaposuchidae are also reconsidered after an exhaustive revision of the morphological data. New and improved diagnoses for each species are here provided.

## Introduction

**Fig 1:**
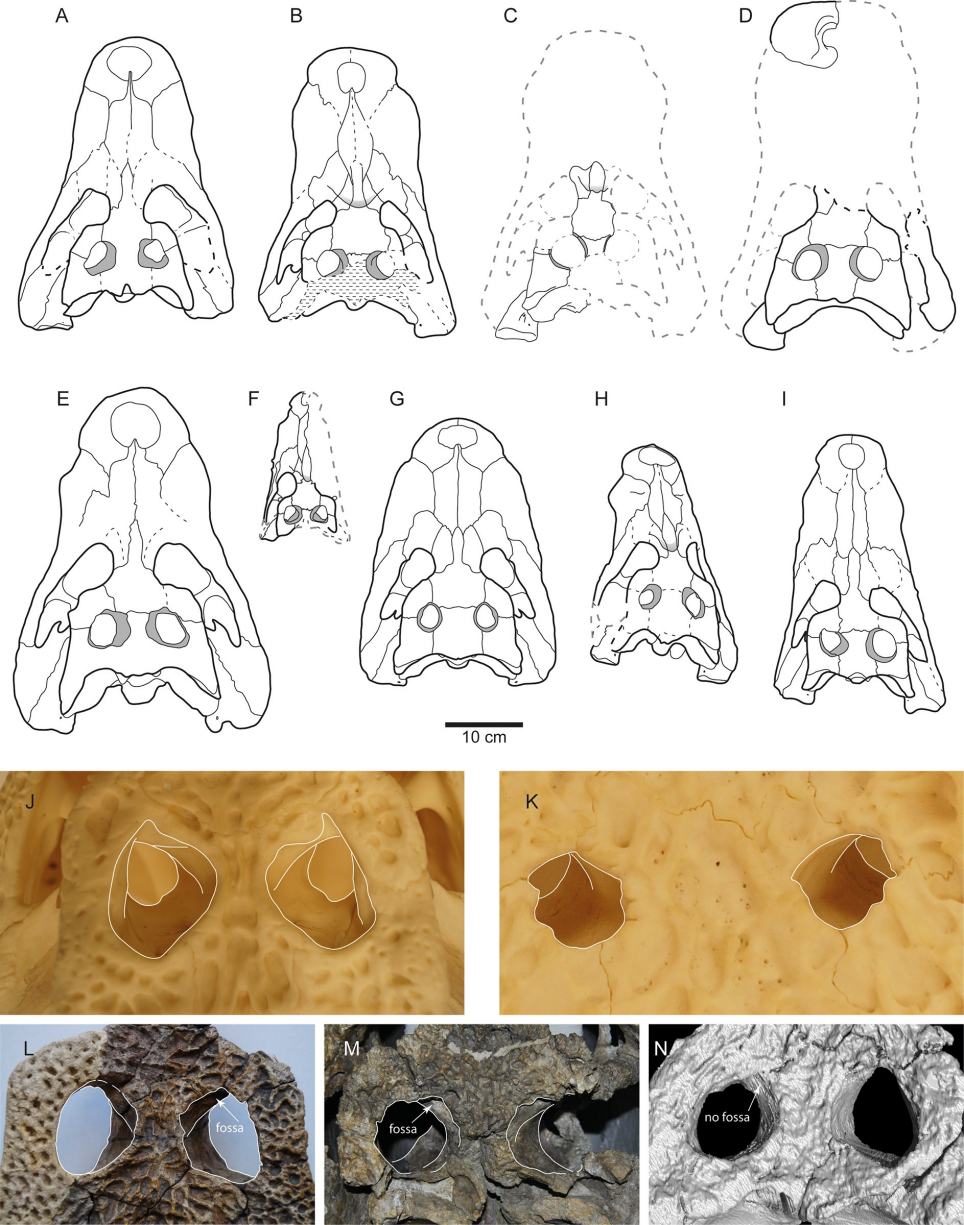
Skulls of allodaposuchid taxa in dorsal view. **A)** *Allodaposuchus precedens*. **B)** *Allodaposuchus subjuniperus*. **C)** *Allodaposuchus palustris*. **D)** *Allodaposuchus hulki*. **E)** *Allodaposuchus iberoarmoricanus*. **F)** *Arenysuchus gascabadiolorum*. **G)** ‘*Lohuecosuchus*’ *megadontos*. **H)** ‘*Lohuecosuchus*’ *mechinorum*. **I)** ‘*Agaresuchus*’ *fontisensis*. **J-N)** Detail of the supratemporal fossa in *Crocodylus niloticus* (J), *Osteolaemus tetraspis* (K), *Allodaposuchus precedens* (L), *Allodaposuchus subjuniperus* (M), and *Allodaposuchus hulki* (N). Fig M, courtesy of Dr. Eduardo Puértolas Pascual. Fig N is a detail of the CT-scan of *Allodaposuchus hulki*, courtesy of Dr. Josep Fortuny.

*‘Allodaposuchus’* is probably the most emblematic eusuchian from the Campanian and Maastrichtian of Europe due to its broad distribution along the archipelago during the end of the Cretaceous [1, 2]. However, its history is not exempt of controversy. *Allodaposuchus precedens* Nopcsa 1928 was described at the beginning of the past century on the basis of fragmentary cranial and postcranial material from the Densus Ciula Formation at Vălioara (Transylvania, Romania) [3, 4]. More recently, Buscalioni et al. [5] reviewed the material from Romania and described new fragmentary remains from Spain (Armuña, Vilamitjana and Laño localities) and France (Bellevue locality), which they referred to the same species. Later, Delfino et al. [6] reported a complete skull from Oarda de Jos (Romania) that they referred to *A*. *precedens* (Fig 1A). After relating the new discovery to the material from Vălioara, the authors suggested that the former *Allodaposuchus* remains from western Europe could be distinguished at the species level from *A*. *precedens*.

In the last years, new skulls and partial skeletons referable to *Allodaposuchus* or closely related forms have been recovered from several sites in France and Spain [7–13] leading to a hotspot in the research focused on the taxonomy, diversity, phylogenetic relationships and palaeobiogeography of European eusuchians. In this sense, three new species were described from different units in the Tremp Formation (northeastern Spain): *Allodaposuchus subjuniperus* Puértolas-Pascual, Canudo & Moreno-Azanza 2014 (Fig 1B), from the fluvial settings of Serraduy (Huesca); *Allodaposuchus palustris* Blanco, Puértolas-Pascual, Marmi, Vila & Sellés 2014 (Fig 1C), from the brackish coastal-palustrine settings of the Fumanya Sud site (Barcelona); and *Allodaposuchus hulki* Blanco, Fortuny, Vicente, Luján, García-Marçà & Sellés 2015 (Fig 1D), from the ephemeral-pond settings of Casa Fabà site (Lleida). These two latter taxa are the only specimens with enough associated postcranial bones. Subsequently, Narváez et al. [11, 12] erected three other allodaposuchid species: *Lohuecosuchus megadontos* Narváez, Brochu, Escaso, Pérez-García & Ortega 2015 (Fig 1G) and *Agaresuchus fontisensis* Narváez, Brochu, Escaso, Pérez-García & Ortega 2016 (Fig 1I) from Lo Hueco (Cuenca, central Spain), as well as *L*. *mechinorum* Narváez, Brochu, Escaso, Pérez-García & Ortega 2015 (Fig 1H) from Fox-Amphoux (southern France), increasing the generic diversity.

In contrast, Martin et al. [13] referred two new skulls (Fig 1E) and five fragmentary cranial remains from the Velaux-La Bastide Neuve site (France) to *Allodaposuchus precedens*. These authors defended that *A*. *precedens* is the only valid species of *Allodaposuchus*, arguing that purported differences between *A*. *precedens*, *A*. *subjuniperus*, *A*. *palustris* and *Lohuechosuchus megadontos* (*L*. *mechinorum* and *Agaresuchus* were not described at that moment) reflect pathology or insufficient preservation in the Spanish specimens, or fall within the purported intraspecific variability range of *Allodaposuchus precedens*. In addition, Narváez et al. [11, 12] also questioned the validity of *A*. *palustris* and *A*. *hulki* due to their fragmentary nature and did not include these taxa in their phylogenetic analyses. However, these studies did not provide morphological evidences for invalidating these taxa besides alluding to their fragmentary nature. Accordingly, Blanco & Brochu [14] recently revised the morphological variability amongst allodaposuchids, demonstrating that diagnostic characters supporting these allodaposuchid species do not fall into the range of intraspecific variability and therefore *A*. *palustris* and *A*. *hulki* must not be considered conspecific to any other allodaposuchid taxa; whereas the material reported by Martin and collaborators likely represents another different taxon from *A*. *precedens*, and was referred to an indeterminate allodaposuchid. The latter study provided an exhaustive morphological discussion, contributing to the knowledge of intra- and interspecific variability on the European allodaposuchids, distinguishing a vast taxonomic diversity in the European archipelago. Additional works [15–18] reported different chronological occurrences and palaeoenvironmental segregation between allodaposuchid species, explaining such taxonomic diversity.

Simultaneously to the advance on the *Allodaposuchus* taxonomy, several studies remarked that three other fossil eusuchians–*Massaliasuchus affuvelensis* (Matheron 1869) Martin & Buffetaut 2008, *Arenysuchus gascabadiolorum* Puértolas, Canudo & Cruzado-Caballero 2011, and *Musturzabalsuchus buffetauti* Buscalioni, Ortega & Vasse 1997 –could be more closely related to *Allodaposuchus* than originally thought [19–21]. *Arenysuchus* was initially considered a basal crocodyloid [20]. However, subsequent phylogenetic analyses recovered *Arenysuchus* as the sister taxa of *Allodaposuchus* [7, 10] grouped in Allodaposuchia. On the other hand, *Massaliasuchus* is based on poorly-preserved material and *Musturzabalsuchus* is only represented by some dentaries and maxillae, so, although their proximity to *Allodaposuchus* has been suggested through descriptive approaches, these taxa are usually excluded from the phylogenetic analyses due to the lack of enough anatomical information [12].

Another important controversy is the phylogenetic emplacement of *Allodaposuchus* and its relatives. The classical approaches proposed a basal eusuchian position (1) as sister taxon of Crocodylia [5]; (2) included within the family Hylaeochampsidae [6, 22, 23]; or (3) as the sister taxon of Hylaeochampsidae [9]. But all of these hypotheses are exclusively based on cranial characters: none of the studied specimens included associated postcranial remains. After the inclusion of postcranial features in the phylogenetic analyses, this viewpoint changed [7, 10], pointing to a shift to a more derived position within Crocodylia. Later, Martin et al. [13] defended again the inclusion of *Allodaposuchus* within Hylaeochampsidae based on a phylogenetic analysis with a much reduced number of taxa and lacking any other allodaposuchid species besides ‘*Allodaposuchus precedens’*. Similarly, Narváez et al. regarded allodaposuchids as sister taxa of hylaeochampsids firstly [11], but then placed Allodaposuchidae as sister taxa of Crocodylia [12]. Nevertheless, these three latest analyses are again based on cranial remains exclusively. Likewise, another controversial conclusion of the phylogenetic analyses performed by Narváez et al. [11, 12] is the statement that the genus *Allodaposuchus* only includes the species *A*. *precedens*. These authors argued that *A*. *precedens* (and therefore the genus) is restricted to eastern European specimens whereas the occurrences in western Europe should be reclassified in new genera. Accordingly, they relocated *A*. *subjuniperus* into the genus *Agaresuchus* [12]. However, this hypothesis lies once again in a phylogenetic analysis exclusively based on cranial remains, with the exclusion of *A*. *palustris* and *A*. *hulki* from the analyses.

Therefore, the phylogenetic status of allodaposuchids with respect to other eusuchian taxa and their internal relationships seem still unresolved, far from reaching a consensus, and pending of the inclusion of postcranial information in the phylogenetic analyses. Hence, the aims of the present work are to test the effects of the postcranial skeleton in the phylogeny, to clarify the phylogenetic position of Allodaposuchidae in Eusuchia, and to reassess the phylogenetic relationships amongst allodaposuchids including all known species in the analysis for first time, providing a revised diagnosis for each one.

## Materials and methods

In contrast to the phylogenetic analyses performed by Blanco et al. [7, 10] based on the dataset provided by Brochu [23], this study works on the data matrix used by Narváez et al. [12], which is in turn mainly based on that of Brochu & Storrs [24]. In the new analyses of the present paper, three new taxa were added to the dataset: *Allodaposuchus palustris*, *A*. *hulki* and the indeterminate allodaposuchid from Velaux-La Bastide Neuve site (coding based on the complete adult skull MMS/VBN-12-10A exclusively; Fig 1E). The whole dataset resulted in 108 OTUs, and the non-eusuchian taxon *Goniopholis* was chosen as the outgroup. A total of 189 morphological characters were compiled using Mesquite 3.31 [25]. The data matrix includes 38 postcranial skeletal characters (numbers 1–37 and 189), 11 characters concerning dermal shield (numbers 38–46, 183 and 184) and 140 cranial characters (numbers 47–182 and 185–188). All characters were treated as unordered and equally weighted. The character matrix was analysed using maximum parsimony (traditional search method) in TNT 1.5 [26, 27]. Heuristic searches with 1000 random replicates and tree-bisection-reconnection branch swapping were performed, holding 10 most parsimonious trees at each step. Strict consensus trees were calculated (S1 Dataset). Bootstrap frequencies (1,000 bootstrap replicates searched) were used to assess the robustness of the nodes.

### Testing the effects of postcranial information on phylogeny and relationships with other eusuchians

**Fig 2:**
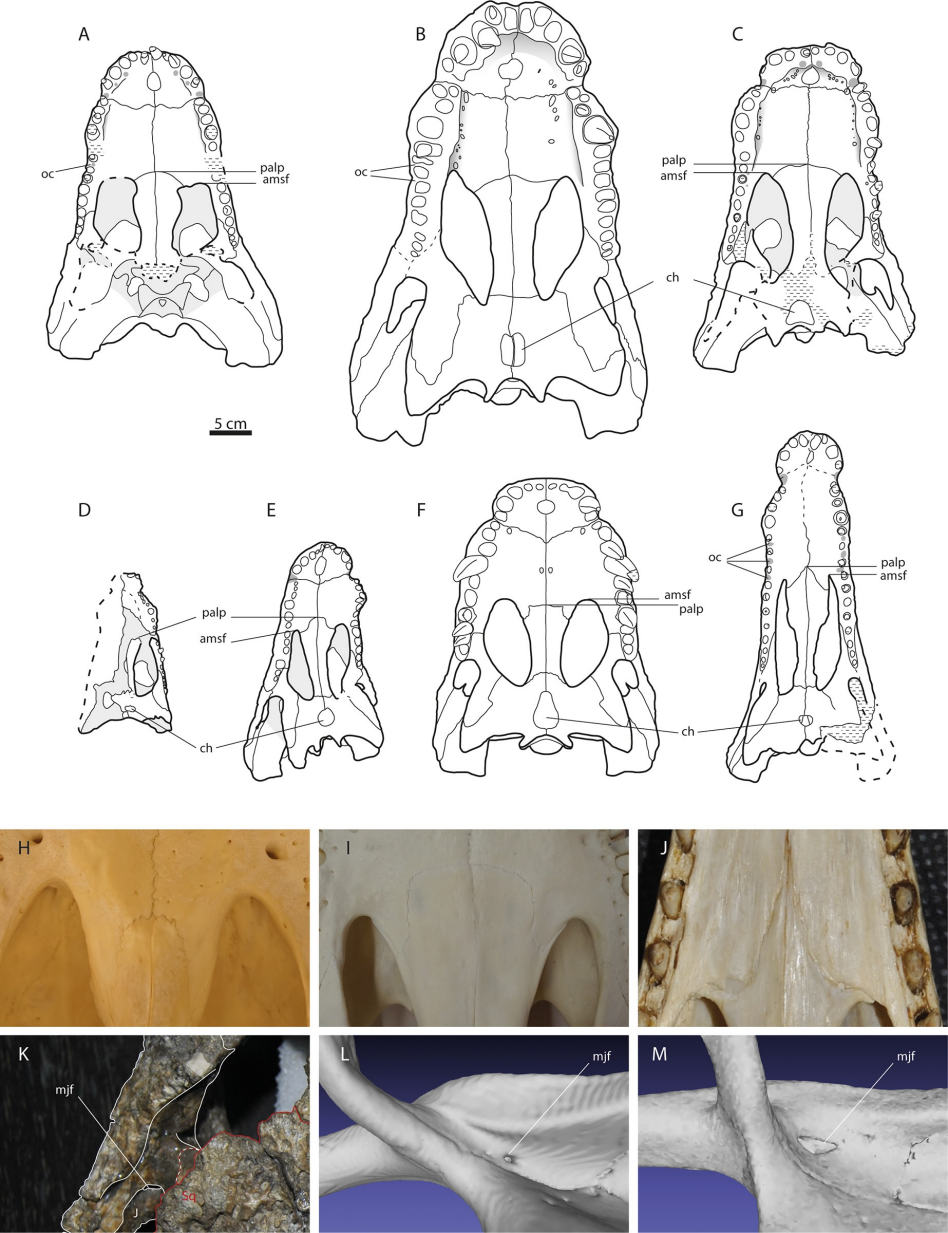
Skulls of allodaposuchid taxa in palatal view. **A)** *Allodaposuchus precedens*. **B)** *Allodaposuchus iberoarmoricanus*. **C)** *Allodaposuchus subjuniperus*. **D)** *Arenysuchus gascabadiolorum*. **E)** ‘*Lohuecosuchus*’ *mechinorum*. **F)** ‘*Lohuecosuchus*’ *megadontos*. **G)** ‘*Agaresuchus*’ *fontisensis*. **H-J)** Detail of the palatine processes in *Osteolaemus tetraspis* (H), *Alligator mississippiensis* (I), and *Mecistops cataphractus* (J). **K-M)** Detail of the jugal and the medial jugal foramen in *A*. *subjuniperus* (K), *Alligator* (L) and *Tomistoma* (M). Fig J and K, courtesy of Dr. Eduardo Puértolas Pascual. Fig N and M are details of CT-scans, courtesy of Dr. Josep Fortuny.

In order to assess the changes related to postcranial information, the first phylogenetic analyses hold the character scorings used in the most recent hypothesis [12]. Through conservative analyses, any change in the tree topology with respect those previously reported should be consequence of the new, postcranial features in the matrix. Hence, scorings from the former analysis of Narváez et al. [12] have been kept, except for some unequivocal wrong scores disagreeing with the anatomical descriptions reported by Narváez et al. [11, 12] themselves, and other authors [6, 9, 20] (Figs 1 and 2).

In *Arenysuchus*, characters 79 and 93 were changed to states 0 and 1 respectively, because the alveoli and preserved teeth in the skull clearly display a circular cross-section, and the largest maxillary alveolus is obviously the fifth [20] (Fig 2D). Character 132 was scored with state 0 because the ectopterygoid reaches the postorbital bar [20]. Character 155 was changed to state 0 because quadrate separates parietal and squamosal on the posterior wall of the supratemporal fenestra [20].

For *Allodaposuchus precedens*, character 131 was changed to state 0 because the frontal ends in an acute tip anteriorly [6] (Fig 1A).

In *Allodaposuchus subjuniperus*, characters 124 and 125 were changed to state 0 because it lacks notched and septate internal choana [9] (Fig 2C). As described by Narváez et al. [12], *Allodaposuchus subjuniperus* differs from *Agaresuchus fontisensis* in lacking a septate internal choana. However, this character has not been scored accordingly for *A*. *subjuniperus* in that dataset. Character 132 was also scored with state 0 in *Allodaposuchus subjuniperus* because the ectopterygoid reaches the postorbital bar [9]. The lateral carotid foramen opens laterally to basisphenoid in *A*. *subjuniperus* (character 169^0^) [9], but dorsally in *Agaresuchus fontisensis* [12]. However, this character has not been scored adequately for *A*. *subjuniperus* in the latter dataset [12]. Thus, character 169 was changed to state 0 accordingly.

In *Lohuecosuchus mechinorum*, characters 124 and 125 were also changed to state 0, because it also lacks notched and septate internal choana [11] (Fig 2E).

In *Agaresuchus fontisensis*, the prootics are not visible [12], showing the same condition as in *Lohuecosuchus* (character 164^1^). However, these taxa have been scored with states 0 and 1 respectively in the dataset [12]. Therefore, character 164 was changed to state 1 in *Agaresuchus* accordingly, because the prootics are not exposed externally.

**Fig 3:**
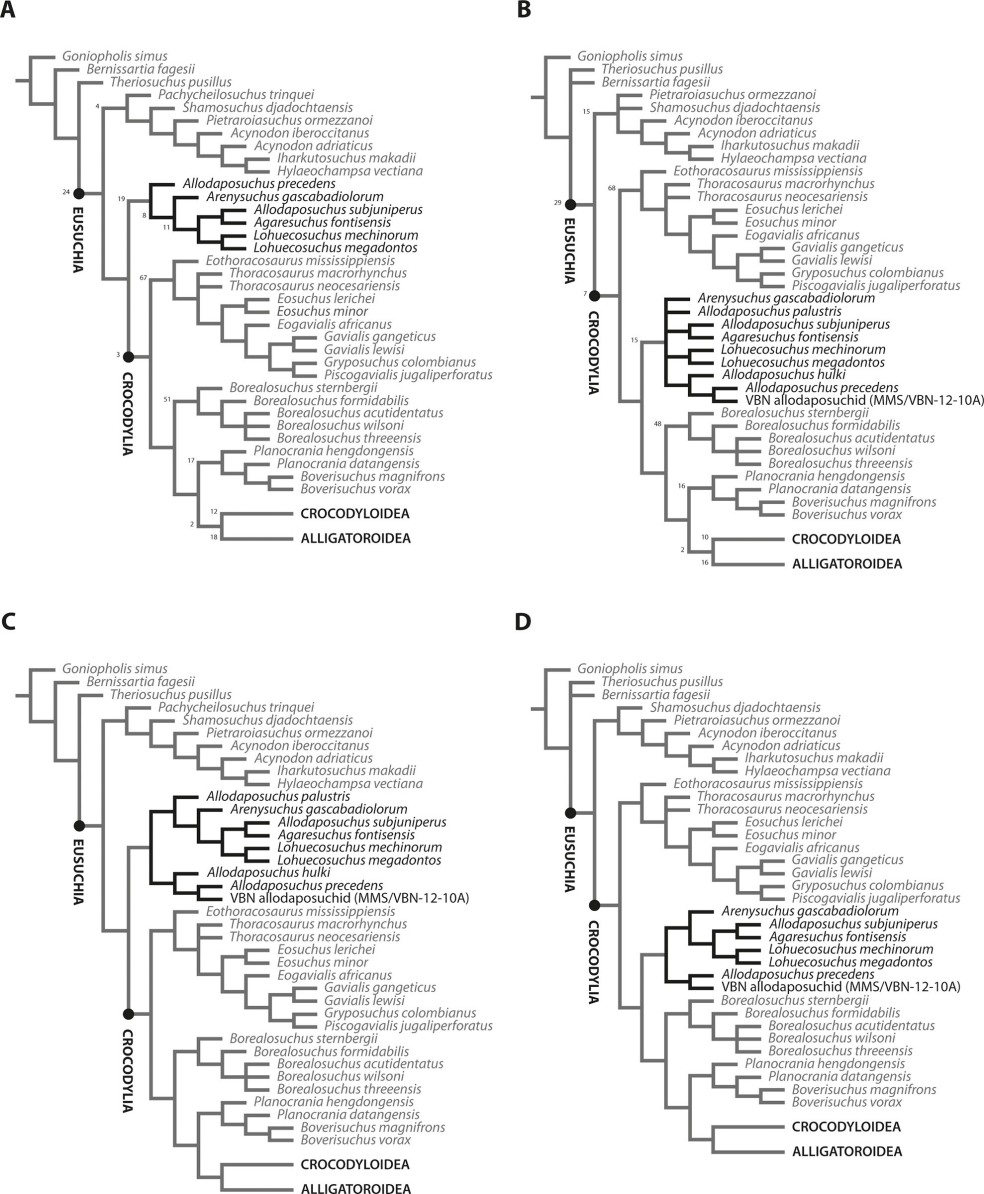
Comparison between phylogenetic analyses including and lacking postcranial information: **A)** reproduction of the analysis of Narváez et al. [12]; **B)** addition of *A*. *palustris* and *A*. *hulki* with postcranial information to the dataset of Narváez et al. [12]; **C)** topology from replicated phylogenetic analysis excluding postcranial information in allodaposuchid taxa; and **D)** topology from phylogenetic analysis including postcranial information, but lacking *A*. *palustris* and *A*. *hulki* as OTUs. Numbers indicate bootstrap support for the main nodes.

After these changes, the phylogenetic analysis on which this study is based on was reproduced as a control (Fig 3A), in order to preliminary check potential changes on the tree topology derived from those recodifications.

Subsequently, three new taxa were added to the dataset: *Allodaposuchus palustris*, *A*. *hulki* and the indeterminate allodaposuchid from Velaux-La Bastide Neuve site (based on the complete adult skull MMS/VBN-12-10A exclusively). Scores for *A*. *palustris* and *A*. *hulki* have been revised: the frontoparietal suture (character 151) of *A*. *palustris* was described as concavoconvex by Blanco et al. [7], but this character has been criticised in subsequent works [11–13]. It was suggested that such character was probably contributing to place allodaposuchids in the derived position recovered in the phylogeny reported by Blanco et al. [7, 10]. Therefore, despite still agreeing with the original description, this character was scored here as missing datum (?) for *A*. *palustris*, in order to not mask the effects of the postcranial information.

A set of three consecutive phylogenetic analyses were performed in order to test the effects of the postcranial information in the phylogeny. The first one was run including all the species, with all cranial and postcranial characters (Fig 3B; S1 Dataset). A second analysis was reproduced under the same parameters, but excluding all postcranial information in the allodaposuchid taxa (Fig 3C). For this purpose, a modified matrix has been built with missing data for all the postcranial characters in every allodaposuchid species (S2 Dataset). By doing so, I tested whether the changes in the phylogenetic position of Allodaposuchidae respect to that reported in previous studies (Fig 3A) are related to the addition of the postcranial information or to the inclusion of *A*. *palustris* and *A*. *hulki* in the dataset. In the third analysis, the same postcranial features observed in *A*. *palustris* and *A*. *hulki* were assumed for the other members of Allodaposuchidae, but excluding these two taxa from the analysis as single OTUs (Fig 3D). This way, the changes in the phylogenetic relationships derived exclusively from the postcranial characters of allodaposuchids are evaluated from the viewpoint of those studies that did not recognize *A*. *palustris* and *A*. *hulki* as valid species [11–13]. Thus, the postcranial features known from the fossil remains referred to *A*. *palustris* and *A*. *hulki* were included in other allodaposuchid taxon with unknown postcranial remains (e.g., *Lohuecosuchus*) of the original matrix.

### Reconstructing the phylogenetic relationships within Allodaposuchidae

A final phylogenetic analysis was performed in order to assess the relationships amongst allodaposuchians. The phylogenetic hypotheses recently reported were not based on all the allodaposuchid taxa [11–13, 28], but after the increase in the European Late Cretaceous crocodyliform diversity that has taken place in recent years [7–14, 18, 20, 28, 29], a review of this clade is necessary.

**Fig 4:**
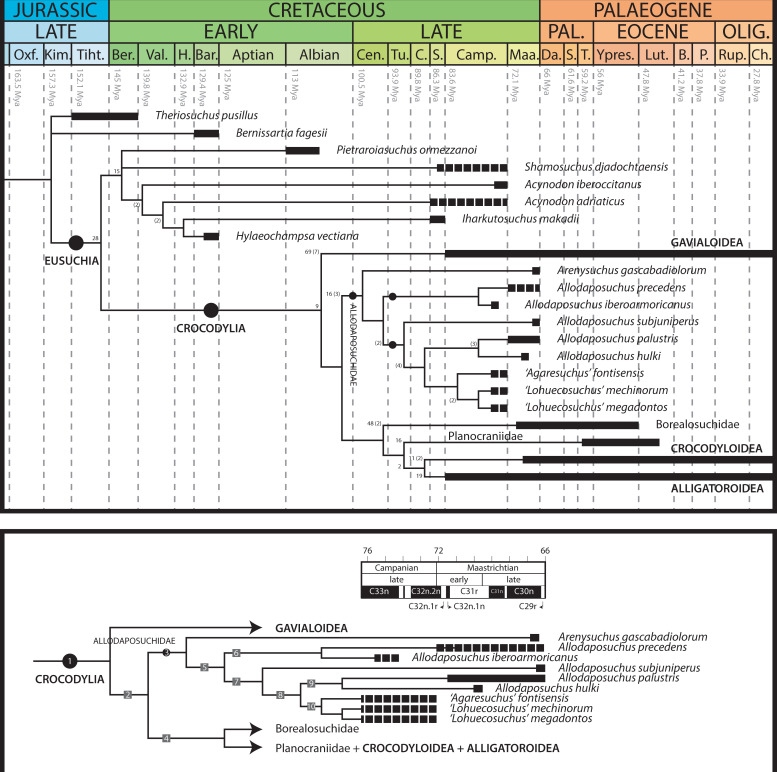
Strict consensus tree of the phylogenetic analysis with revised character codification and postcranial information (up), CI: 0.29, RI: 0.77; and detail of the family Allodaposuchidae showing common synapomorphies (down). Numbers in the consensus tree (up) indicate bootstrap frequencies for the main nodes, and Bremer support (over 2) in parentheses. Chronological ranges according to Fondevilla et al. [55]. Discontinuous bars represent approximate chronological ranges, and continuous bars reflect well-calibrated occurrences. Node 1 (Crocodylia): characters 71^1^, 74^0^, 118^1^, 121^1^, 131^0^, 158^1^; node 2: characters 12^1,^ 28^1^ 135^1^; node 3 (Allodaposuchidae): characters 128^1^, 137^1^, 148^0^, 149^0^, 151^1^, 153^0^; node 4: characters 63^2^, 108^1^, 125^1^, 173^1^; node 5: characters 102^1^; node 6: character 92^1^; node 7: characters 87^1^, 133^0^; node 8: characters 142^2^, 152^1^; node 9: character 153^1^; node 10: characters 73^1^, 102^0^, 169^1^.

This latter analysis includes several recodifications in some characters according with own observations (Fig 4; S3 Dataset):

Narváez et al. [12] considered that dermal bones of the skull roof (character 152) overhang the rims of the supratemporal fenestra in all allodaposuchids (state 1), but this is not true for *Arenysuchus*, *A*. *precedens* and *A*. *subjuniperus* (state 0), as also noted by Delfino et al. [6], Puértolas-Pascual et al. [9] and Martin et al. [13]. In these taxa, the postorbital, parietal and squamosal do not project over the supratemporal fossa (Fig 1), as it is in *Crocodylus niloticus* (Fig 1J) rather than in *Osteolaemus tetraspis* (Fig 1K). Therefore, the character 152 was scored accordingly with state 0 for *Allodaposuchus precedens* (Fig 1A and 1L) (and see below for *Arenysuchus* and *A*. *subjuniperus*).

Character 115, related to the extension of the palatine process, is better regarded as state 1 in *Arenysuchus* according to the original description of the character by Brochu [30]. In this taxon, the palatine process does not extend beyond the anterior end of suborbital fenestra. Character 142 was scored as missing datum in *Arenysuchus* due to the preservation of the posterior angle of the supratemporal fenestra [20]. As said above, dermal bones of the skull roof do not overhang the rims of the supratemporal fenestra in *Arenysuchus* (Fig 1F), so character 152 was scored with state 0 accordingly.

In *Allodaposuchus subjuniperus*, character 102 was scored with state 1, because a large medial jugal foramen clearly appears posteriorly to the postorbital (Fig 2K). As the palatine process extends beyond the anterior end of the suborbital fenestra (Fig 2C), the character 115 is better scored with state 0 in this taxon, following the description of the character [30]. This condition is similar to that of *Alligator mississippiensis* (Fig 2I) rather than to that showed in *Osteolaemus tetraspis* (Fig 2H). As stated above, dermal bones of the skull roof do not overhang the rims of the supratemporal fenestra in *A*. *subjuniperus* (Fig 1B and 1M), the character 152 was scored accordingly with state 0 for this taxon.

For *Lohuechosuchus mechinorum*, the character 115 was also changed to state 0 following the description of Brochu [30]. In this taxon, the palatine process also extends beyond the anterior end of suborbital fenestra (Fig 2E).

In *Agaresuchus fontisensis*, the character 55 was scored as state 1, because the coronoid completely surrounds the *foramen intermandibularis medius* [12]; the character 115 was also changed to state 0 (Fig 2G) according to the original description of the character [30]; and character 116 was scored as state 1 because the palatine process ends in a thin wedge, the same condition as shown in *Mecistops cataphractus* (Fig 2J).

It should be noted that anterior palatine processes of *Lohuechosuchus mechinorum*, *Agaresuchus* and *A*. *subjuniperus* extend beyond the anterior margin of the suborbital fenestra (character 115^0^), the same condition that *A*. *precedens* shows. In spite of Narváez et al. [11, 12] recognized this similarity, the authors scored this character differently in *A*. *precedens* and in the other species without further explanation. Furthermore, these authors stated that *A*. *precedens* is the only allodaposuchid with a large medial jugal foramen (character 102^1^) [11, 12]. However, the same feature had been previously described for *A*. *subjuniperus* and *A*. *hulki* [9, 10] (Fig 2K–2M). Such considerations may have led the authors to distinguish *A*. *precedens* from the Iberian taxa, but these purported exclusive features of *A*. *precedens* are not actually justified.

Finally, the recodifications of *Iharkutosuchus* and changes in the characters 92, 118, 119, 128, 152, 158, 167, 170, 173 for *Allodaposuchus precedens*, *A*. *subjuniperus* and *Arenysuchus* proposed by Mateus et al. [28] were also included (see detailed explanations and figures in their Supporting information). However, concerning dental occlusal patterns (character 92), *Allodaposuchus precedens* shows a “semi in-line” occlusion with interalveolar pits between the 6^th^ and the 7^th^ maxillary teeth [6, 28], whereas *Agaresuchus fontisensis* shows interalveolar pits between the 6^th^ and the 9^th^ positions [12]. Both conditions differ from each other, and from those in other allodaposuchids with an overbite occlusion pattern (Fig 2). Therefore, *A*. *precedens* and *Agaresuchus* are better regarded as having states 1 and 2, respectively, in order to reflect these differences.

### Nomenclatural acts

The electronic edition of this article conforms to the requirements of the amended International Code of Zoological Nomenclature, and hence the new names contained herein are available under that Code from the electronic edition of this article. This published work and the nomenclatural acts it contains have been registered in ZooBank, the online registration system for the ICZN. The ZooBank LSIDs (Life Science Identifiers) can be resolved and the associated information viewed through any standard web browser by appending the LSID to the prefix “http://zoobank.org/”. The LSID for this publication is: urn:lsid:zoobank.org:pub:1BFE8D82-43CB-4F65-8382-B86CC7AF33A2. The electronic edition of this work was published in a journal with an ISSN, and has been archived and is available from the following digital repositories: PubMed Central, LOCKSS.

## Results

The reproduction of the analysis of Narváez et al. [12] results in 1247 most-parsimonious trees of 783 steps, and delivered the same strict consensus topology they reported, even after the little re-scoring that was performed (Fig 3A). This fact evidences that subsequent changes of the tree topology in further analyses should be related to additional taxa including postcranial information.

The first phylogenetic analysis, where *A*. *palustris*, *A*. *hulki* and the allodaposuchid from Velaux-La Bastide Neuve were included (S1 Dataset), results in 3690 most-parsimonious trees of 797 steps. Although relations amongst allodaposuchids are not fully resolved, the clade was recovered as member of Crocodylia: more closely related to *Borealosuchus*, planocraniids and the crown-group Brevirostres than to gavialoids (Fig 3B). This change in the phylogenetic position may be a consequence of the inclusion of these three taxa in the analysis, or alternatively, caused by the postcranial information associated with two of them. In order to clarify the question, the test was re-run (second analysis) including these taxa again, but deleting codifications in their postcranial characters (S2 Dataset). This analysis results in 1158 most-parsimonious tress of 798 steps, and the resulting topology of the consensus tree (Fig 3C) places allodaposuchids in a basal position, similarly to that reported in previous hypotheses [12]. Furthermore, the relationships between allodaposuchids are fully resolved. This fact points to the postcranial information as the reason for the shift in the phylogeny, since all the allodaposuchid taxa were included in this analysis, and when only the cranial characters are analysed the clade is recovered outside Crocodylia. Likewise, if *A*. *palustris* and *A*. *hulki* are not included in the analysis as additional OTUs, but their postcranial scores are included in the dataset as common features of allodaposuchids, the consensus tree resulting from the third analysis (4132 most-parsimonious trees of 784 steps) shows again the shift in the position grouping them in Crocodylia (Fig 3D).

The final phylogenetic test including several re-scorings in the revised characters results in 3020 trees of 805 steps (Fig 4). Allodaposuchids are placed in Crocodylia, more related to *Borealosuchus* and the other crocodylians than to gavialoids. The clade of allodaposuchids is fully resolved, as in the analysis based on cranial characters exclusively (Fig 3C). *Arenysuchus* is recovered apart from the other allodaposuchian representatives. The indeterminate allodaposuchid from Velaux-La Bastide Neuve appears as the sister taxon of *A*. *precedens*. *Agaresuchus fontisensis* is recovered as the sister taxon of *Lohuecosuchus*. *Allodaposuchus hulki* is grouped with *A*. *palustris*, as the sister clade of *Agaresuchus + Lohuecosuchus*. However, *Allodaposuchus subjuniperus* is placed as a stem taxon related to the node including *A*. *palustris*, *A*. *hulki*, *Lohuecosuchus* and *Agaresuchus*. A few previous hypotheses regarded *A*. *subjuniperus* as a member of *Agaresuchus* [12, 28]. However, the current results do not support such referral because *A*. *subjuniperus* is set apart from the type species of the genus *Agaresuchus*. Based on this analysis, the genus *Agaresuchus sensu* Narváez et al. [12] is polyphyletic. Moreover, the genus *Allodaposuchus* as currently known is paraphyletic, because some of its representatives (*A*. *palustris*, *A*. *hulki* and *A*. *subjuniperus*) share a more recent common ancestor with *Lohuecosuchus* and *Agaresuchus* rather than to *A*. *precedens*. In other words, *Lohuecosuchus* and *Agaresuchus* are nested in the node grouping all the other species referred to the genus *Allodaposuchus*. This means that *Allodaposuchus* would be paraphyletic while *Lohuecosuchus* and *Agaresuchus* are considered different genera. Therefore, the needed for a taxonomic review becomes evident.

## Discussion

### Relationships between allodaposuchids and other eusuchians

Characters supporting the position of allodaposuchids as members of Crocodylia are the following unambiguous synapomorphies: retroarticular process projected posterodorsally (C71^1^); absence of sulcus between articular and surangular (C74^0^); palatine-pterygoid suture far from posterior angle of suborbital fenestra (C118^1^); choana surrounded by pterygoids (C121^1^); anterior tip of frontal forms simple acute point (C131^0^); significant posterolateral squamosal rami along paroccipital process (C158^1^). In contrast, the clade formed by *Pietraroiasuchus* and hylaeochampsids is distinguished by equal anterior processes of surangular (C61^1^); fourth dentary tooth occludes in a pit between premaxilla and maxilla (C91^1^); maxilla with posterior process between lacrimal and prefrontal (C128^2^); and reduced or absent quadratojugal spine (C140^1^), placed between posterior and superior angles of infratemporal fenestra (C141^1^). Additionally, allodaposuchids differentiate from gavialoids and are grouped with *Borealosuchus* and the other crocodylians according to the following unambiguous synapomorphies: uncrested axial neural spine (C12^1^); *M*. *teres major* and *M*. *dorsalis scapulae* insert with common tendon on humerus (C28^1^); and maxillary tooth row curves laterally broadly posterior to first six maxillary alveoli (C135^1^).

Furthermore, allodaposuchids (at least *A*. *hulki*) share a narrow and sub-angular olecranon process of the ulna (C29^0^) with *Borealosuchus*. As *A*. *hulki* is the only taxon preserving the ulna, this character currently shows an ambiguous evolutionary history. However, TNT reconstructed five equally-parsimonious hypothesis among which one proposes this character as a synapomorphy shared between allodaposuchids and *Borealosuchus* (but posteriorly reverted in planocraniids and Brevirostres). The addition of further postcranial information to the dataset might clarify if the feature can be finally considered an unambiguous synapomorphy or not.

As demonstrated by the three consecutive phylogenetic analyses (Fig 3), the inclusion of Allodaposuchidae within the crown-group of Crocodylia is a consequence of the addition of postcranial information to the dataset. Remarkably, a basal phylogenetic position in Eusuchia is only recovered when the analyses are completely based on cranial characters (Fig 3A and 3C). The phylogenetic emplacement of allodaposuchids shifts when postcranial characters are considered, regardless *A*. *palustris* and *A*. *hulki* are recognized or not (Fig 3B and 3D). The results reported in the present paper evidence the relevance of the postcranial skeleton in phylogenetic approaches. These results also support the phylogenetic hypothesis for Allodaposuchia reported by Blanco et al. [7, 10], despite being based on different datasets, and differ from other studies carried out with specimens only known by cranial remains [6, 9, 11–13, 28]. As members of Crocodylia, the stratigraphic record of allodaposuchids in the late Campanian and Maastrichtian of Europe is not incongruent with that of other crocodylians. The earliest confirmed records of recognized crocodylians are even older, belonging to alligatoroids and gavialoids from the early Campanian of North America and Europe [2, 31, 32].

It is commonly thought that the crocodyliform skeleton is morphologically conservative throughout their evolutionary history [32–34]. Thus, our current knowledge on the crocodyliform evolution, and especially for Eusuchia, is mainly based on the skull morphology. The postcranial anatomy of crocodyliforms is usually neglected in many works, and most taxonomic descriptions are based exclusively on skulls. Intriguingly, this fact does not seem related to the nature of the findings because fossil specimens are frequently found with associated postcranial remains; but they are excluded from the studies or merely described superficially [5, 11–13, 19, 21, 22, 35]. Likewise, the current morphological phylogenetic datasets are strongly biased towards the cranial anatomy, and the postcranial skeleton only represents 12.9–22.5% of the scored characters [23, 24, 36, 37] or is simply absent [38, 39]. Strikingly, the highest representation of the postcranial skeleton in a phylogenetic dataset (31.2%) was performed by Norell & Clark [40], who included 5 (of 16 total) characters in their analysis. This strong bias towards the skull morphology also promotes circularly numerous studies on crocodylomorph systematics made with cranial information only, accumulating a large number of taxa with missing data on postcranial characters in datasets. However, the crocodyliform lifestyle ranges from fully terrestrial to semiaquatic and fully marine forms, and it is logical to expect that postcranial skeletons reflect ecological adaptations. Although the most extreme specializations to different lifestyles occurred in other clades (e.g., Thalattosuchua, Notosuchia) and the eusuchian anatomy is mainly constrained by semiaquatic habits, cursorial forms and differences in locomotor patterns have been also identified amongst crocodylians [39, 41–49]. In this sense, some studies found significant morphological differences under the apparent phenotypic conservativeness of the skeleton [46–48, 50], and evidenced that postcranial features may contribute with their own phylogenetic signal [51]. The results of the phylogenetic analyses here reported are in agreement with this latter statement, demonstrating that phylogenetic hypotheses might change when the postcranium is included in the dataset.

### Relationships amongst allodaposuchids

Allodaposuchids are a monophyletic clade supported by the following unambiguous synapomorphies (Fig 4): maxilla with posterior process within lacrimal (C128^1^); upturned dorsal edges of orbits (C137^1^); caudal margin of otic aperture not defined and gradually merging into the exoccipital (C148^0^); quadrate and squamosal not in contact on the external surface of the skull, posteriorly to the external auditory meatus (C149^0^); linear frontoparietal suture (C151^1^) (reverted in *A*. *palustris* [7, 14]); presence of shallow fossa at anteromedial corner of supratemporal fenestra (C153^0^) (reverted in *A*. *palustris* and *A*. *hulki*). The clade subsequently splits in *Arenysuchus* and the other allodaposuchids, grouped by a large medial jugal foramen (C102^1^) (as ancestral condition posteriorly reverted in some taxa). Among these taxa, *Allodaposuchus precedens* and the allodaposuchid from Velaux-La Bastide Neuve split from the rest of Iberian taxa. *Allodaposuchus precedens* and the indeterminate allodaposuchid are grouped by the semi-inline occlusion pattern (C92^1^). The other Ibero-Armorican taxa are grouped by the presence of four premaxillary teeth (C87^1^) (posteriorly reverted in *Lohuecosuchus*) and a massive postorbital bar (C133^0^) (posteriorly reverted in *Lohuecosuchus megadontos*). Among those Ibero-Armorican allodaposuchids, *A*. *hulki* and *A*. *palustris*, and *Agaresuchus fontisensis* and *Lohuecosuchus* are sister taxa, respectively, and more derived than *A*. *subjuniperus*. Unambiguous synapomorphies supporting the node *Lohuecosuchus + Agaresuchus* are: surangular-articular suture bowing strongly laterally (C73^1^); small medial jugal foramen (C102^0^); and lateral carotid foramen opens dorsally to basisphenoid (C169^1^). On the other hand, *A*. *hulki* and *A*. *palustris* share a smooth anteromedial corner of supratemporal fenestra (absence of shelf) (C153^1^) (Fig 1N). The node including *A*. *hulki*, *A*. *palustris*, *Agaresuchus fontisensis* and *Lohuecosuchus* is supported by the quadratojugal-jugal suture that lies at posterior angle of infratemporal fenestra (C142^2^); and dermal bones of skull roof overhanging the rim of supratemporal fenestra (C152^1^).

Special mention deserves the absence of mandibular fenestra in allodaposuchids. Mandibular fenestra appeared and was lost several times throughout the crocodylomoph evolution, and its reappearance has been sometimes interpreted as a feature distinguishing Crocodylia from basal eusuchians and other neosuchians [30, 36, 40]. Accordingly, the absence in allodaposuchids was argued to relate this clade to basal eusuchians [11, 12]. Thus, it could be expected that the absence of mandibular fenestra (C63^0^) appears as an autapomorphy of Allodaposuchidae if allodaposuchids are members of Crocodylia. However, the current phylogenetic hypothesis suggests a very different scenario: strikingly, the absence of the mandibular fenestra seems to be a symplesiomorphic feature for Crocodylia, which opened independently in gavialoids and derived crocodylians. This is congruent with observations describing the lack or a reduced fenestra in several fossil crocodylians (e.g., *Borealosuchus*, *Portugalosuchus*, *Deinosuchus*, *Mekosuchus*) and some gavialoids [22, 28, 52].

In their conclusions, Narváez et al. [11, 12] regarded *Allodaposuchus palustris* and *A*. *hulki* as invalid species because of their fragmentary preservation, and these taxa were not included in the phylogenetic analyses they performed. The resulting phylogenetic topology led the authors to consider *A*. *subjuniperus* as a member of the genus *Agaresuchus*, and to restrict the genus *Allodaposuchus* to the species *A*. *precedens*, occurring exclusively in eastern Europe. However, the phylogenetic hypothesis proposed in the present study shows significant discrepancies. Firstly, the phylogenetic positions of *A*. *palustris* and *A*. *hulki* are fully resolved (Fig 4), even when the analysis of Narváez et al. [12] was reproduced including them (Fig 3C). These taxa show both autapomorphies and a unique combination of synapomorphies that allow to differentiate them from other allodaposuchid taxa. Therefore, the statement of these taxa are undiagnosable and too fragmentary for phylogenetic analyses has no support. Moreover, the split between ‘eastern’ and ‘western’ European allodaposuchids reported in previous hypotheses [11, 12] are not supported by the results of the present paper (Fig 4), because the French indeterminate allodaposuchid is closely related to *A*. *precedens* in all the analyses (Figs 3 and 4). This fact, together with the taxonomic validity of *A*. *palustris* and *A*. *hulki*, argues against the restriction of the genus *Allodaposuchus* to the Romanian specimens.

As stated above, the genus *Agaresuchus*, as defined by Narváez et al. [12] is polyphyletic. After the revised scores (Fig 4; see also Mateus et al., [28]) *Allodaposuchus subjuniperus* has not been recovered as sister taxa of *Agaresuchus fontisensis*. Likewise, *Allodaposuchus–*as currently known–would be paraphyletic if *Agaresuchus* and *Lohuecosuchus* are considered as different genera, because these latter taxa are nested among other species referred to *Allodaposuchus*. This raises two possibilities: (1) either considering *Agaresuchus* and *Lohuecosuchus* as junior synonyms of *Allodaposuchus*, or (2) rename several stem taxa (i.e., *A*. *subjuniperus*, *A*. *hulki* and *A*. *palustris*) into additional monospecific genera in order to restrict the genus *Allodaposuchus* to a monophyletic clade including the type species and its closely-related taxon. In this work, big taxonomical changes will be avoided and the species referred to the genera *Agaresuchus* and *Lohuecosuchus* must be considered as members of *Allodaposuchus*. However, further studies might change this viewpoint.

### Systematic Palaeontology

The inclusion of all the allodaposuchid taxa in a phylogenetic analysis, here for first time, may change the taxonomic diagnoses published in previous studies. Therefore, the diagnoses for these taxa should be revised and improved, based on the results of the cladistics analysis herein reported. These diagnoses are based on the unambiguous synapomorphies and autapomorphies identified for each taxon after the analysis. Additionally, characters shared between some taxa are discussed in the differential diagnoses, providing a unique combination of characters for each species.

Order CROCODYLIFORMES Hay 1930

Unranked MESOEUCROCODYLIA Whestone & Whybrow 1983

Suborder EUSUCHIA Huxley 1875

Unranked CROCODYLIA Gmelin 1789

Family ALLODAPOSUCHIDAE Narváez et al. 2015

**Type species:** *Allodaposuchus precedens* Nopcsa 1928.

**Referred species:** *Allodaposuchus precedens*, *Allodaposuchus iberoarmoricanus* sp. nov., *Allodaposuchus subjuniperus*, *Allodaposuchus hulki*, *Allodaposuchus palustris*, ‘*Agaresuchus*’ *fontisensis*, ‘*Lohuecosuchus*’ *megadontos*, ‘*Lohuecosuchus*’ *mechinorum*, and *Arenysuchus gascabadiolorum*.

**Definition:** see Narváez et al. [11].

**Emended diagnosis:** The clade is characterized by the following six autapomorphies: maxilla with posterior process within lacrimal; upturned dorsal edges of orbits; caudal margin of otic aperture not defined and gradually merging into the exoccipital; quadrate and squamosal not in contact on the external surface of the skull, posteriorly to the external auditory meatus; linear frontoparietal suture (reverted in *A*. *palustris*); presence of shallow fossa at anteromedial corner of supratemporal fenestra (reverted in *A*. *palustris* and *A*. *hulki*).

**Remarks:** The original definition [11] also include *Massaliasuchus affuvelensis* and *Musturzabalsuchus buffetauti*. However, there are no evidences supporting these referrals and alternative hypotheses for the phylogenetic relationships of these taxa cannot be confidently rejected [19, 21]. Therefore, their inclusion in Allodaposuchidae is still pending further analyses.

Previous works [11, 12] also listed the following characters as diagnostic for Allodaposuchidae: the tenth dentary alveolus is the largest behind the fourth in the mandibular tooth row; dermal bones of skull roof overhanging the rim of the supratemporal fenestra; ventral margin of the postorbital bar being part of the lateral jugal surface; nasals contacting external naris, but not bisecting it; and the fourth maxillary alveolus being the largest in the tooth row. Nevertheless, these features should not be considered diagnostic for allodaposuchids. Such relation between nasals and the external naris is an ambiguous autapomorphy shared with hylaeochampsids, planocraniids and most crocodyloids and alligatoroids. The insertion of the postorbital bar in the jugal surface is a common feature to all crocodylians except gavialoids. On the other hand, the largest maxillary alveolus in the fourth position occurs in derived allodaposuchids, but it is the fifth maxillary position in *Arenysuchus* (a plesiomorphic state according to the current analyses); whereas the position of the largest dentary alveolus varies among allodaposuchid taxa. Moreover, the condition of the dermal bones of the skull roof was wrongly interpreted in those works (see other descriptions [6, 9, 20, 28] and Material & Methods).

Genus *ARENYSUCHUS* Puértolas et al. 2011

**Type species:** *Arenysuchus gascabadiolorum* Puértolas et al. 2011.

**Diagnosis:** as for the species (monospecific genus).

*Arenysuchus gascabadiolorum* Puértolas et al. 2011

**Description:** see Puértolas et al. [20].

**Emended diagnosis:** Allodaposuchid whose largest maxillary alveolus is the fifth; and short palatine process which does not extend beyond the anterior end of suborbital fenestra.

**Differential diagnosis:** Additionally, *Arenysuchus* can be differentially distinguished from other allodaposuchids by the unique combination of the following features: dermal bones of skull roof do not overhang rim of the supratemporal fenestra (shared with *Allodaposuchus precedens*, *A*. *iberoarmoricanus* sp. nov. and *A*. *subjuniperus*), frontal with a low transverse interorbital ridge at the beginning of the anterior process (shared with *Allodaposuchus subjuniperus* and *A*. *palustris*, and unlike *Allodaposuchus precedens*, *Allodaposuchus iberoarmoricanus* sp. nov., ‘*Agaresuchus*’ *fontisensis*, ‘*Lohuecosuchus*’ *megadontos* and *Allodaposuchus hulki*), small medial jugal foramen (shared with ‘*Lohuecosuchus*’ and ‘*Agaresuchus*’ *fontisensis*), lateral carotid foramen opens laterally to basisphenoid (unlike ‘*Agaresuchus*’ *fontisensis* and ‘*Lohuecosuchus*’), and presence of shallow fossa at anteromedial corner of supratemporal fenestra (unlike *Allodaposuchus hulki* and *A*. *palustris*).

Genus *ALLODAPOSUCHUS* Nopcsa 1928

**Type species:** *Allodaposuchus precedens* Nopcsa 1928

**Referred species:** *Allodaposuchus precedens*, *Allodaposuchus iberoarmoricanus* sp. nov., *Allodaposuchus subjuniperus*, *Allodaposuchus hulki*, *Allodaposuchus palustris*, ‘*Agaresuchus*’ *fontisensis*, ‘*Lohuecosuchus*’ *megadontos*, and ‘*Lohuecosuchus*’ *mechinorum*.

**Emended diagnosis:** Allodaposuchid with large medial jugal foramen (reverted in ‘*Agaresuchus*’ *fontisensis* and ‘*Lohuecosuchus*’) and the largest maxillary alveolus in the fourth position.

*Allodaposuchus precedens* Nopcsa 1928

**Description:** see Nopcsa [4] and Delfino et al. [6].

**Emended diagnosis:** Species of *Allodaposuchus* showing a pit between premaxilla and maxilla for the occlusion of the fourth dentary tooth (unique among allodaposuchids), semi-inline occlusal pattern (shared with *A*. *iberoarmoricanus*), ornamented tooth enamel with parallel and continuous apicobasal ridges, and lacking septate internal choana.

**Remarks:** The medial jugal foramen opens anteriorly to the postorbital bar (unlike *A*. *iberoarmoricanus*). Also, the capitate process of laterosphenoid is here considered laterally oriented [see also 6, 11, 12]. This condition differ from that in all other allodaposuchids, likely including *A*. *iberoarmoricanus* sp. nov., although this feature cannot be confidently assessed in this latter [13]. Thus, the lateral orientation of the capitate process of laterosphenoid could be regarded autapomorphic of *A*. *precedens*.

Additionally, some authors [11, 12] also regarded the anteroposteriorly wide basisphenoid, exposed as a broad sheet ventrally to the basioccipital (characters 172 and 173) and the large medial jugal foramen as exclusive autapomorphies for *A*. *precedens*. Although an anteroposteriorly wide basisphenoid (character 172^1^) was not described in other allodaposuchids, the current analysis recovered it as an ambiguous apomorphy. The occipital exposition of the basisphenoid ventrally to the basioccipital was misunderstood in previous studies [11, 12]: this feature was scored correctly in the dataset, but wrongly described as a broad exposition. The basisphenoid is not broadly exposed ventrally to the basioccipital in *A*. *precedens*, as in *A*. *iberoarmoricanus* sp. nov., *A*. *subjuniperus* and ‘*L*.’ *mechinorum* [11, 13, 28]. On the other hand, a large medial jugal foramen had been described in *A*. *subjuniperus* and *A*. *hulki* [9, 10].

Finally, the nasals are abruptly attenuated (constrained) towards external naris. This condition differs with most allodaposuchids, but it is shared with ‘*L*.’ *megadontos* and *A*. *iberoarmoricanus*.

*Allodaposuchus iberoarmoricanus* sp. nov.

urn:lsid:zoobank.org:act:1642302B-F8C7-4E6E-A1FE-A41611C7CE97

**Holotype:** MMS/VBN-12-10A.

**Etymology:** Refers to the distribution of this taxon in the Ibero-Armorican island, in the Cretaceous European Archipelago.

**Locality and horizon:** Velaux-La Bastide Neuve, Bouches du Rhône Department, southern France. Fluvial deposits from the Late Campanian.

**Description:** see Martin et al. [13].

**Diagnosis:** Species of *Allodaposuchus* with internal choana with septum (that remains recessed within choana), semi-inline occlusal pattern and tooth enamel ornamented with parallel and continuous apicobasal ridges (as *A*. *precedens*). Additionally, the dentary bears sixteen tooth positions, being the eleventh the largest alveolus (unlike ‘*Agaresuchus*’ *fontisensis*, ‘*Lohuecosuchus*’ and *A*. *palustris*).

**Remarks:** A large medial jugal foramen is shared with *A*. *precedens*, *A*. *subjuniperus* and *A*. *hulki*. However, the medial jugal foramen opens posteriorly to the postorbital bar (unlike *A*. *precedens*). Furthermore, Martin et al. [13] noted a strange arrangement of the three first maxillary teeth, which they considered anomalous. Despite not considering this character here as diagnostic for this taxon, further studies might determine whether this feature could be regarded an additional autapomorphy for the species or simply pathologic.

*Allodaposuchus subjuniperus*

**Description:** see Puértolas-Pascual et al. [9]

**Emended diagnosis:** *Allodaposuchus* with naris oriented dorsally (as in ‘*Agaresuchus*’ *fontisensis*), palatine-maxillary suture intersects suborbital fenestra at its anteriormost limit (shared with ‘*L*.’ *mechinorum*).

**Differential diagnosis:** Additionally, *A*. *subjuniperus* can be distinguished from other allodaposuchids by the combination of the following symplesiomorphies: four premaxillary teeth (unlike *Allodaposuchus precedens*, *A*. *iberoarmoricanus* sp. nov. and ‘*Lohuecosuchus’*), unornamented dental enamel (unlike *A*. *precedens*, *A*. *iberoarmoricanus* sp. nov. and *A*. *palustris*), quadratojugal forming posterior angle of infratemporal fenestra (shared with *A*. *precedens*, *A*. *iberoarmoricanus* sp. nov., *Arenysuchus* and ‘*L*.’ *mechinorum*), basisphenoid not broadly exposed ventral to the basioccipital (unlike ‘*Agaresuchus*’ *fontisensis* and ‘*L*.’ *megadontos*), dermal bones of skull roof do not overhang the rim of the supratemporal fenestra (unlike *A*. *hulki*, *A*. *palustris*, ‘*Agaresuchus*’ *fontisensis* and ‘*Lohuecosuchus*’), frontal with a low transverse interorbital ridge at the beginning of the anterior process (shared with *Arenysuchus* and *A*. *palustris*), a massive postorbital bar (unlike *A*. *precedens*, *A*. *iberoarmoricanus* sp. nov., *Arenysuchus* and ‘*L*.’ *mechinorum*), a large medial jugal foramen (unlike *Arenysuchus*, ‘*Agaresuchus*’ *fontisensis* and ‘*Lohuecosuchus*’) which opens posteriorly to the postorbital bar (unlike *A*. *precedens* and *A*. *hulki*), and lateral carotid foramen opens laterally to basisphenoid (unlike ‘*Agaresuchus*’ *fontisensis* and ‘*Lohuecosuchus*’).

*Allodaposuchus hulki*

**Description:** see Blanco et al. [10].

**Emended diagnosis:** *Allodaposuchus* with absence of quadratojugal spine, and absence of shallow fossa at anteromedial corner of supratemporal fenestra (shared with *A*. *palustris*).

**Differential diagnosis:** Additionally, *A*. *hulki* can be distinguished from other allodaposuchids by the combination of the following features: naris opens anterodorsally (unlike ‘*Agaresuchus*’ *fontisensis* and *A*. *subjuniperus*), four premaxillary teeth (unlike *A*. *precedens*, *A*. *iberoarmoricanus* sp. nov. and ‘*Lohuecosuchus*’), unornamented dental enamel (unlike *A*. *precedens*, *A*. *iberoarmoricanus* sp. nov. and *A*. *palustris*), large medial jugal foramen (unlike *Arenysuchus*, ‘*Agaresuchus*’ *fontisensis* and ‘*Lohuecosuchus*’) which opens anteriorly to the postorbital bar (unlike *A*. *iberoarmoricanus* and *A*. *subjuniperus*), quadratojugal-jugal suture lies at posterior angle of infratemporal fenestra (unlike *A*. *precedens* and *A*. *iberoarmoricanus* sp. nov., and shared with ‘*Lohuecosuchus*’ and ‘*Agaresuchus*’ *fontisensis*), frontal without transverse interorbital ridge at the beginning of the anterior process (unlike *Arenysuchus*, *A*. *subjuniperus*, and *A*. *palustris*), and dermal bones of skull roof overhanging the rim of supratemporal fenestra (unlike *Arenysuchus*, *Allodaposuchus* and *A*. *subjuniperus*).

**Remarks:** Both quadrate hemicondyles are similar in size and the medial hemicondyle is not ventrally deflected. This condition differs from that in all other allodaposuchids, except in a juvenile skull from Velaux-La Bastide Neuve site [13]. Therefore, it can be considered a paedomorphic feature retained in matures *A*. *huki* [14], becoming apomorphic for this taxon.

*Allodaposuchus palustris*

**Description:** see Blanco et al. [7].

**Emended diagnosis:** *Allodaposuchus* lacking the shallow fossa at anteromedial corner of supratemporal fenestra (shared with *A*. *hulki*), exoccipital without boss on paroccipital process, large quadrate *foramen aëreum*, teeth with ornamented rugose enamel showing abundant and divergent small ridges developing false-ziphodont carinae.

**Differential diagnosis:** Additionally, *A*. *palustris* can be distinguished from other allodaposuchids by the combination of the following features: lateral carotid foramen opens laterally to basisphenoid (unlike ‘*Agaresuchus*’ *fontisensis* and ‘*Lohuecosuchus*’), dermal bones of skull roof overhanging the rim of supratemporal fenestra (unlike *Arenysuchus*, *A*. *precedens*, *A*. *iberoarmoricanus* sp. nov. and *A*. *subjuniperus*), frontal with a low transverse interorbital ridge at the beginning of the anterior process (unlike *Allodaposuchus precedens*, *A*. *iberoarmoricanus* sp. nov. and ‘*Agaresuchus*’ *fontisensis*), dentary with thirteen tooth positions (unlike ‘*Agaresuchus*’ *fontisensis*, ‘*Lohuecosuchus*’ and *Allodaposuchus iberoarmoricanus* sp. nov., and unknown in other allodaposuchids), the largest dentary alveolus is the ninth (unlike ‘*Agaresuchus*’ *fontisensis*, ‘*Lohuecosuchus*’ and *Allodaposuchus iberoarmoricanus* sp. nov., and unknown in other allodaposuchids), surangular-articular suture oriented anteroposteriorly (not bowed), and equal anterior processes of surangular (unlike ‘*Agaresuchus*’ *fontisensis* and ‘*Lohuecosuchus*’, and unknown in other allodaposuchids).

*Allodaposuchus fontisensis* Narváez et al. 2016

**Description:** see Narváez et al. [12].

**Emended diagnosis:** Allodaposuchid with naris projected dorsally (shared with *A*. *subjuniperus*), consecutive interalveolar pits between the sixth to the ninth tooth positions, palatine process form a thin wedge anteriorly (V-shaped), lateral edges of palatines with lateral process projecting into suborbital fenestrae, and internal choana with septum (shared with *A*. *iberoarmoricanus* sp. nov.).

**Differential diagnosis:** Additionally, *A*. *fontisensis* can be distinguished from other allodaposuchids by the combination of the following symplesiomorphies: four premaxillary teeth (unlike *A*. *precedens*, *A*. *iberoarmoricanus* sp. nov. and ‘*Lohuecosuchus*’), unornamented dental enamel (unlike *A*. *precedens*, *A*. *iberoarmoricanus* sp. nov. and *A*. *palustris*), frontal without transverse interorbital ridge at the beginning of the anterior process (unlike *Arenysuchus*, *A*. *subjuniperus* and *A*. *palustris*), surangular-articular suture strongly bowed laterally (unlike *A*. *palustris* and shared with ‘*Lohuecosuchus*’ *megadontos*), small medial jugal foramen (unlike *A*. *precedens*, *A*. *iberoarmoricanus* sp. nov., *A*. *subjuniperus* and *A*. *hulki*), lateral carotid foramen opens dorsally to basisphenoid (unlike *Arenysuchus*, *Allodaposuchus precedens*, *A*. *iberoarmoricanus* sp. nov., *A*. *subjuniperus* and *A*. *palustris*), jugal forming posterior angle of infratemporal fenestra (shared with ‘*Lohuecosuchus*’ and *A*. *hulki*), basisphenoid exposed as broad sheet ventral to basioccipital (only shared with ‘*L*.’ *megadontos*), and dermal bones of skull roof overhanging the rim of supratemporal fenestra (shared with ‘*Lohuecosuchus*’, *A*. *palustris* and *A*. *hulki*).

**Remarks:** *A*. *fontisensis* and *A*. *subjuniperus* were grouped in the genus ‘*Agaresuchus*’ based on the dorsally-projected external naris and in the premaxillary dental series composed by only four tooth position [12]. Nevertheless, four premaxillary alveoli are also present in *A*. *hulki*; whereas the dorsally-projected naris seems to be an autapomorphy independently adquired in both *A*. *fontisensis* and *A*. *subjuniperus*.

In addition, the emplacement of the quadratojugal-jugal suture in the posterior angle of infratemporal fenestra was described as an autapomorphy of *A*. *fontisensis* [12]. However, the same condition was also observed in *A*. *hulki* [10] and ‘*L*.’ *megadontos* [11].

*Allodaposuchus megadontos* Narváez et al. 2015

**Description:** see Narváez et al. [11]

**Emended diagnosis:** Allodaposuchid showing a naris wider than long (shared with ‘*L*.’ *mechinorum*), five premaxillary teeth (shared with *A*. *precedens*, *A*. *iberoarmoricanus* sp. nov. and ‘*L*.’ *mechinorum*), prominent preorbital ridges (shared with ‘*L*.’ *mechinorum*), straight pterygoid ramus of ectopterygoid making a linear posterolateral margin of suborbital fenestra (shared with ‘*L*.’ *mechinorum*), premaxillary surface with a deep notch lateral to naris, prominent *canthi rostralii*, nasals abruptly attenuated (constrained) towards external naris (shared with *A*. *precedens* and *A*. *iberoarmoricanus* sp. nov.), short palatine process which does not extend beyond the anterior end of suborbital fenestra (shared with *Arenysuchus*), and slender postorbital bar (shared with *Arenysuchus*, *A*. *precedens* and *A*. *iberoarmoricanus* sp. nov.).

**Differential diagnosis:** Additionally, *A*. *megadontos* can be distinguished from other allodaposuchids by the combination of the following symplesiomorphies: small medial jugal foramen (unlike *A*. *precedens*, *A*. *iberoarmoricanus* sp. nov., *A*. *subjuniperus* and *A*. *hulki*), and lateral carotid foramen opens dorsally to basisphenoid (unlike *Arenysuchus*, *A*. *precedens*, *A*. *iberoarmoricanus* sp. nov., *A*. *subjuniperus* and *A*. *palustris*), quadratojugal-jugal suture lies at posterior angle of infratemporal fenestra (shared with *A*. *fontisensis* and *A*. *hulki*), and dermal bones of skull roof overhanging the rim of supratemporal fenestra (unlike *Arenysuchus*, *Allodaposuchus precedens*, *A*. *iberoarmoricanus* sp. nov. and *A*. *subjuniperus*, but shared with *A*. *fontisensis*, *A*. *palustris* and *A*. *hulki*).

*Allodaposuchus mechinorum* Narváez et al. 2015

**Description:** see Narváez et al. [11]

**Emended diagnosis:** Allodaposuchid with a naris wider than long (shared with ‘*L*.’ *megadontos*), five premaxillary teeth (shared with *A*. *precedens*, *A*. *iberoarmoricanus* sp. nov. and ‘*L*.’ *megadontos*), prominent preorbital ridges (shared with ‘*L*.’ *megadontos*), straight pterygoid ramus of ectopterygoid making a linear posterolateral margin of suborbital fenestra (shared with ‘*L*.’ *megadontos*), quadratojugal forming the posterior angle of infratemporal fenestra (shared with *A*. *precedens*, *A*. *iberoarmoricanus* sp. nov., *A*. *subjuniperus* and *Arenysuchus*), and palatine-maxillary suture intersects suborbital fenestra at its anteriormost limit (shared with *A*. *subjuniperus*).

**Differential diagnosis:** Additionally, *A*. *mechinorum* can be distinguished from other allodaposuchids by the combination of the following symplesiomorphies: small medial jugal foramen (unlike *A*. *precedens*, *A*. *iberoarmoricanus* sp. nov., *A*. *subjuniperus* and *A*. *hulki*), and lateral carotid foramen opens dorsally to basisphenoid (unlike *Arenysuchus*, *A*. *precedens*, *A*. *iberoarmoricanus* sp. nov., *A*. *subjuniperus* and *A*. *palustris*), and dermal bones of skull roof overhanging the rim of supratemporal fenestra (unlike *Arenysuchus*, *Allodaposuchus precedens*, *A*. *iberoarmoricanus* sp. nov. and *A*. *subjuniperus*, but shared with *A*. *fontisensis*, *A*. *palustris* and *A*. *hulki*).

**Remarks:** The presence of a massive postorbital bar and the absence of broad exposition of the basisphenoid ventral to the basioccipital (characters 133^0^ and 173^1^) have been proposed as diagnostic for this species [11, 12]. However, the massive postorbital bar is shared by several allodaposuchids (i.e. *A*. *subjuniperus* and *A*. *fontisensis*); whereas the short exposition of the basisphenoid is shared with *A*. *subjuniperus*, *A*. *precedens* and *A*. *iberoarmoricanus* sp. nov. Actually, *A*. *mechinorum* lacks unique features absent in other allodaposuchids.

### Comments on the palaeobiogeography of allodaposuchids

With the description of *Allodaposuchus palustris*, Blanco et al. [7] proposed for first time a palaeobiogeographic model for allodaposuchians based on a S-DIVA analysis. Such hypothesis suggested an Ibero-Armorican origin for the genus *Allodaposuchus*, who dispersed later reaching the Transylvanian island; and finally it underwent a vicariant event giving different species in eastern and western sectors of the European archipelago. It should be noted that vicariance is defined as a biogeographical process implying the split of the continuous geographical distribution of a given ancestral population into, at least, two different parts by means of a geographic barrier [53, 54]. In this way, a population of a widespread species progressively diverges into different subspecies, and species, in the new areas while the ancestor becomes extinct in the intermediate area. Vicariance is one of the mechanism of the allopatric speciation, but it should not be assumed a priori as the sole explanation for allopatric processes [54]. So, the hypothesis proposed by Blanco et al. [7] was supported by the oldest known record of *Allodaposuchus*, reported from the Campanian of Spain and France, and by the widespread occurrence of the genus in the European Archipelago [5, 6].

Later, Narváez et al. [11] also stated that the speciation observed between allodaposuchids could be explained under a vicariant model. However, the phylogenetic hypothesis defended by these authors restricts the genus *Allodaposuchus* to the species *A*. *precedens*, and does not recognize the occurrence of the genus *Allodaposuchus* in Ibero-Armorica. Therefore, this statement is incongruent with the vicariant model, because such scenario lacks the hypothetical ancestral population broadly distributed in Europe that gave place to descendant species in split geographical areas.

**Fig 5:**
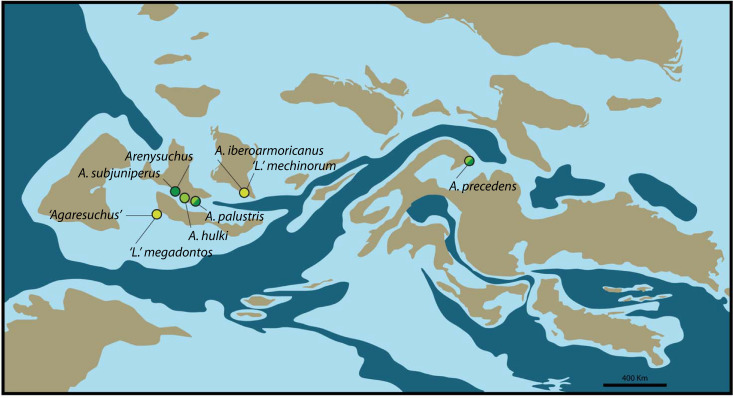
Palaeobiogeographical distribution of Allodaposuchidae. Colours distinguish between late Campanian (yellow), early Maastrichtian (light green) and late Maastrichtian (dark green) taxa. Base map for late Campanian (~ 75 Mya), modified from Csiki-Sava et al. [1] distributed under a CC BY 4.0 license, and based on the reconstruction performed by R. Blakey.

At the light of the current results, the hypothesis of a vicariant event loses even more support. According to the new phylogenetic hypothesis, allodaposuchids seem to have originated in the Ibero-Armorican island, and probably in the Iberian Plate. This is supported by the oldest stratigraphic record and the area of major taxonomic diversity [1, 2, 5, 55]. In this sense, most of the allodaposuchid species remained in the ancestral area of origin (i.e. *Arenysuchus*, *Allodaposuchus subjuniperus*, *Allodaposuchus hulki*, *Allodaposuchus palustris*, *Allodaposuchus fontisensis* and *Allodaposuchus megadontos*); whereas few taxa dispersed to northern geographical areas in the Armorican plate (*A*. *iberoarmoricanus* and *A*. *mechinorum*) or even to the Transylvanian island (*A*. *precedens*) (Fig 5). However, the palaeobiogeographical history of allodaposuchids is obscured by imprecise dating of several sites; and our interpretation might change drastically with alternative phylogenetic hypotheses, as well.

## Conclusions

This study evidences that postcranial bones can contribute with significant phylogenetic signal to the maximum parsimony analyses in crocodylians. Our current hypotheses about eusuchian-crocodyliform evolutionary relationships are primarily determined by skull characters (>77%), thus, strongly biased towards the cranial morphology. However, this is more likely a consequence of the high percentage of missing data in the postcranial information of the dataset, rather than the inability of postcranial features to reflect the evolutionary history of crocodyliforms.

Concerning allodaposuchids, they are recovered as members of Crocodylia when the postcranial information is considered in the phylogenetic analysis, regardless the taxa included in the data matrix. The results support a close relationship with borealosuchids, planocraniids, crocodyloids and alligatoroids, and a more derived phylogenetic position than gavialoids.

After an exhaustive review on character list, data matrix and codification, the phylogenetic relationships among allodaposuchids are reconsidered. The genus *Allodaposuchus* (as known up to day) is recovered paraphyletic if ‘*Agaresuchus*’ and ‘*Lohuecosuchus*’ are considered different genera, because the species referred to these latter genera are nested among other species referred to the genus *Allodaposuchus*. Thus, ‘*Agaresuchus*’ and ‘*Lohuecosuchus*’ must be considered junior synonyms of *Allodaposuchus*. Likewise, Ibero-Armorican specimens from Velaux-La Bastide Neuve (France) previously referred to *A*. *precedens* are better regarded its sister taxon, *Allodaposuchus iberoarmoricanus* sp. nov. The palaeobiogeographic history of the group is still unclear, but they seem to have had an Iberian origin–where most taxa remained–whereas few allodaposuchids dispersed to the Armorican massif and to eastern Europe.

## Supporting information

S1 Dataset Conservative phylogenetic dataset. (TXT)

S2 Dataset Reduced phylogenetic dataset lacking postcranial characters in allodaposuchids. (TXT)

S3 Dataset Revised phylogenetic dataset. (TNT)
